# Molecular Epidemiology of Human Immunodeficiency Virus Type 1 in Guangdong Province of Southern China

**DOI:** 10.1371/journal.pone.0048747

**Published:** 2012-11-07

**Authors:** Song Chen, Weiping Cai, Jingyang He, Nicole Vidal, Chunhui Lai, Weizhong Guo, Haolan He, Xiejie Chen, Linsheng Fu, Martine Peeters, Eric Delaporte, Jean-Marie Andrieu, Wei Lu

**Affiliations:** 1 Sino-French Collaborative Laboratory, Tropical Medicine Institute, Guangzhou University of Chinese Medicine, Guangzhou, China; 2 Laboratoire d’Oncologie et Virologie Moléculaire, Université Paris Descartes, Paris, France; 3 Guangzhou Hospital of Infectious Diseases, Guangzhou, China; 4 UMI 233, Institut de Recherche pour le Développement and Université de Monpellier 1, Monpellier, France; Institut Pasteur of Shanghai, Chinese Academy of Sciences, China

## Abstract

**Background:**

Although the outbreak of human immunodeficiency virus type 1 (HIV-1) in Guangdong has been documented for more than a decade, the molecular characteristics of such a regional HIV-1 epidemic remained unknown.

**Methodology/Principal Findings:**

By sequencing of HIV-1 *pol/env* genes and phylogenetic analysis, we performed a molecular epidemiologic study in a representative subset (n  = 200) of the 508 HIV-1-seropositive individuals followed up at the center for HIV/AIDS care and treatment of Guangzhou Hospital of Infectious Diseases. Of 157 samples (54.1% heterosexual acquired adults, 20.4% needle-sharing drug users, 5.7% receivers of blood transfusion, 1.3% men who have sex with men, and 18.5% remained unknown) with successful sequencing for both pol and env genes, 105 (66.9%) HIV-1 subtype CRF01_AE and 24 (15.3%) CRF07_BC, 9 (5.7%) B’, 5 (3.2%) CRF08_BC, 5 (3.2%) B, 1 (0.6%) C, 3 (1.9%) CRF02_AG, and 5 (3.2%) inter-region recombinants were identified within *pol/env* sequences. Thirteen (8.3%) samples (3 naïves, 6 and 5 received with antiretroviral treatment [ART] 1–21 weeks and ≥24 weeks respectively) showed mutations conferring resistance to nucleoside/nonnucleoside reverse transcriptase inhibitors or protease inhibitors. Among 63 ART-naïve patients, 3 (4.8%) showed single or multiple drug resistant mutations. Phylogenetic analysis showed 8 small clusters (2–3 sequences/cluster) with only 17 (10.8%) sequences involved.

**Conclusion/Significance:**

This study confirms that sexual transmission with dominant CRF01_AE strain is a major risk for current HIV-1 outbreak in the Guangdong’s general population. The transmission with drug-resistant variants is starting to emerge in this region.

## Introduction

Guangdong province, located at the southern coast of China with a registered number of permanent residents reaching 104.3 millions in 2011 (http://www.stats.gov.cn/zgrkpc/dlc/yw/t20110428_402722384.htm), is the first region opened to the world as from 1978. The first HIV-1 case was diagnosed from a traveler infected overseas in 1990. HIV-1 infection was initially confirmed in native intravenous drug users (IDU) in 1996 [1], [2]. Then, the HIV-1 epidemic emerged rapidly in the next 10 years (average 160.3% each year from 1997 to 2007), followed by a significant decrease due to the prevention and control measures taken by the Chinese government [3]. According to the statistics of department of health of Guangdong province, AIDS had been the first factor causing death for consecutive 11 months from March 2009 to January 2010. Although the National Free Antiretroviral Treatment Program” (NFATP) has led a dramatic increase in the national treatment coverage to treatment-eligible individuals (from zero in 2002 to 63.4% in 2009) and significant decrease in the overall mortality (from 39.3 per 100 person-year in 2002 to 14.2 in 2009) [4], the HIV-1-related mortality was as high as 27.9 per 100 person-year patients in Guangdong province in 2009 (http://www.gdwst.gov.cn/a/yiqingxx/201002147510.html). However, it remains unknown whether such a high regional mortality rate of AIDS is caused by a particular HIV-1 outbreak (such as an emergence of new or drug-resistant variants) in Guangdong.

In present study, we conducted a molecular epidemiological investigation in a subset of 508 HIV-1-seropositive individuals followed up from January to September 2009 at the center for AIDS prevention and treatment of Guangzhou Hospital of Infectious Diseases (GHID), the only one authorized for implementing the NFATP program in Guangzhou city (capital of Guangdong province) who had treated around 90% of HIV-1 patients in Guangdong province during the past years.

## Methods

### Participants and Specimens

**Figure 1:**
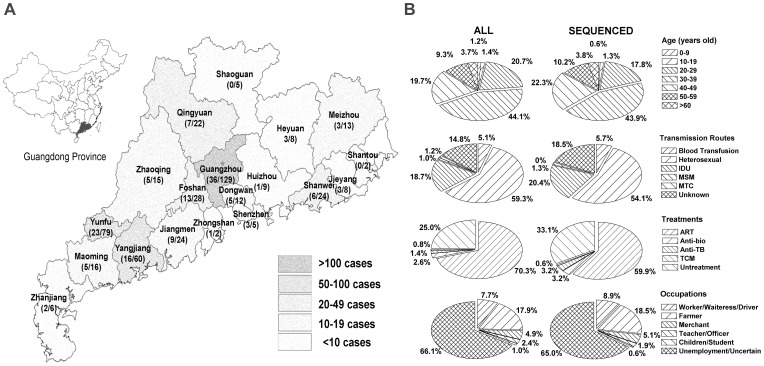
Characteristics of HIV-1 infected individuals in Guangzhou Hospital of Infectious Diseases in 2009. The geographical distribution of 462 patients (Guangdong’s residents) was represented by different levels of gray and the parentheses showed cases of patients enrolled in HIV-1 *pol/env* sequence analysis versus overall patients in this city/county (A). Comparison of characteristics between patients enrolled into the sequence analysis and overall patients, including their age, occupation, risk factors and treatments (B).

From January to September 2009, a total of 508 patients (462 residents from 19/21 cities of Guangdong province and 46 residents from other cities outside of Guangdong) (Fig. 1a) who visited at the center for AIDS prevention and treatment of GHID participated in this study. All patients were required to complete standardized questionnaires (describing sex, age, risk factors, mode of transmission, occupation, geographic location, and treatment, etc) by the national HIV/AIDS surveillance system and sentinel surveillance program [5]. Of 508 patients recruited, 357 (70.3%) cases were currently receiving highly active antiretroviral therapy (HAART). The combinations of antiretroviral drugs included any 2 combinations of 4 NRTIs (Zidovudine [AZT], Didanosine [ddI], Stavudine [d4T] or Lamivudine [3TC], and 1 NNRTIs (Nevirapine or Efavirenz). Twenty patients (3.9%) with tuberculosis (TB) or opportunistic infections (OI) were receiving anti-TB or anti-infection therapy (who were not on HAART and would be followed by free HAART treatment once the TB or OI will be controlled). Finally, 4 patients (0.8%) with a higher CD4 count (>350 cells/µl) received the free Chinese-medicine treatment covered by the national health insurance program.

As the pre-plan design, 200 patients were selected randomly and delivered into the molecular epidemiology and drug resistance survey after consent (written consents were obtained from all patients). The Institutional Ethics Committees of Guangzhou University of Chinese Medicine (GUCM) had approved the study protocol. These investigations have been conducted according to the principles expressed in the Declaration of Helsinki. A total of 5 ml of EDTA-treated whole blood was taken from each patient, and all samples were sent to our laboratory in GUCM. The blood samples were then used immediately for routine blood count and CD4^+^ T-cell count measurements as well as to separate plasma and peripheral blood monocular cells (PBMC). Plasma samples were stored at −80°C while PBMC samples were re-suspended in the storage buffer containing 10% dimethyl sulfoxide (DMSO) (Sigma-Aldrich Corporation, St Louis, Missouri, USA) and 50% fetus bovine serum (Invitrogen Corporation, Grand Island, New York, USA), kept at −80°C for 48 hours, and then stored in liquid nitrogen.

### Viral Load Measurement

All plasma samples were thawed at the same time and were used for viral RNA measurement using the Food and Drug Administration (FDA)-approved Amplicor HIV-1 Monitor Test kit (version 1.5) (Roche Molecular Systems, Inc., Branchburg, New Jersey, USA) according to the manufacturer’s instructions.

### Sequencings of HIV-1 *pol* and *env* Genes

Generally, HIV-1 subtypes, drug resistant mutations, as well as transmission clusters can be detected by sequencing *pol* gene. However, subtypes CRF01_AE and CRF15_01B can only be differentiated by the sequence of *env* region. Moreover, simultaneous sequencing *pol* and *env* genes will be helpful for identify the incidence of potential new inter-subtype recombinants [6], [7], [8]. Initially, viral RNA was extracted from the patients’ plasma (150 µl) using the QIAamp Viral RNA Mini kit (Qiagen, Valencia, California, USA) according to the manufacturer’s instructions. The viral RNA was then subjected to reverse transcription polymerase chain reaction (RT-PCR). The sensitivity and specificity of our amplification system for the different HIV-1 subtypes had been validated previously [9], [10]. By increasing the input sample size (1 ml) concentrated with the use of ultracentrifugation, the detection of limits of our PCR-based sequencing assay could reach 10 copies/ml of HIV-1 RNA. Due to a high occurrence of amplification failure (>50%) even with the use of 1-ml plasma by ultracentrifuge, patient’s PBMC deleted of CD8^+^ cells by antibody-conjugated magnetic beads (Miltenyi Biotec Inc, Auburn, CA, USA) were used for isolating the HIV-1 from all patients. About 100 µl of culture supernatants collected at the peak of viral production were used for HIV-1 RNA extraction. On the basis of the reference sequences obtained from the National Institutes of Health/National Institute of Allergy and Infectious Diseases (NIH/NIAID)-funded HIV database, the genetic subtypes were identified in *pol* gene (1864 base pairs) spanning protease and reverse transcriptase regions and in the V3–V5 region of the *env* gene (660 base pairs) as previously described [7], [11]. The newly obtained sequences were aligned using Muscle [12] as implemented in Seaview v4.3 [13] with reference sequences representing overall HIV-1 group M genetic variability. We included all pure subtypes, sub-subtypes, Asian circulating recombinant forms (CRFs) references (CRF01_AE, CRF07_BC, CRF08_BC, CRF15_01B, CRF33_01B, CRF34_01B, CRF48_01B and CRF51_01B), and also some other CRFs prevalent elsewhere but reported to circulate sometimes at low level in Asia, such as CRF02_AG and CRF13_cpx. Phylogenetic analysis was first conducted for each new sequence individually. The *pol* and *env* fragments, were investigated for recombination using Simplot version 3.5.1 software [14]. Genotyping data were used to identify minor and major resistance mutations in protease and reverse transcriptase genes, based on the last updated online Stanford Resistance Database tool: HIVdb program-Genotypic Resistance Interpretation Algorithm (version 6.1.0, http://sierra2.stanford.edu/sierra/servlet/JSierra? action = sequenceInput). Additionally, in accordance with the latest WHO recommendations, resistance mutations were also evaluated with the last updated list for surveillance of transmitted drug-resistant strains in untreated patients (ver.6.0 http://cpr.stanford.edu/cpr.cgi).

### Phylogenetic Reconstruction

Phylogenetic inter-relationships among viral sequences were estimated using PHYML with the minimal number of reference sequences, i.e excluding the CRFs not represented among the samples of the present study [15], [16]. Phylogenies were inferred using a general-time reversible model of nucleotide substitution, an estimated proportion of invariant sites, and gamma distributed rates among sites. The best of SPR and NNI heuristic options was selected to search the tree space, and bootstrap values with 1000 replicates were used to assess confidence in topology. The existence of transmission clusters was determined using the statistical robustness of the maximum likelihood topologies assessed by high bootstrap values (>98%) with 1000 resamplings and short branch lengths (genetic distances <0.015) of HIV-1 *pol* gene sequence [6], [7].

### Statistical Analysis

Differences in the CD4^+^ T-cell count and viral load among different subgroups of patients were determined using a Mann–Whitney nonparametric test.

### Accession Numbers

All sequences analyzed in the present study are deposited in EMBL under the accession numbers HE590887 to HE591043 and HE591086 to HE591242 for the *pol* and *env* sequences, respectively.

## Results

### Characteristics of HIV-1 Patients in Guangdong

Of 508 HIV-1 seropositive individuals followed up at the center for AIDS prevention and treatment of GHID during January–September 2009 (Fig. 1a), the majority of them were male (315 cases, 62.0%), 20–49 years old (429 cases, 84.5%), unemployment (336 cases, 66.1%), infected by heterosexual contact (301 cases, 59.3%), and currently treated with HAART (357 cases, 70.3%) (Fig. 1b). Among 193 HIV-1-infected women, 152 (78.8%) acquired the virus by heterosexual contact and only 4 (2.1%) were injection drug users (IDUs).

### Viral loads and CD4^+^ T-cell Counts

The mean (±SD) of CD4 counts and the geometric mean (± SE) of viral loads in the 508 patients were 284±213 cells/µl and 5.36±2.66 log_10_ copies/ml respectively. Significant higher viral loads were observed in the 127 (25%) untreated (7.27±1.89 log_10_ copies/ml) than in the 357 (70.3%) HAART-treated (4.49±174 log_10_ copies/ml) patients (P<0.01) while similar CD4 counts were observed between untreated (293±122 cells/µl) and HAART-treated (283±213 cells/µl) patients (P>0.05). The highest viral load (8.89±3.23 log_10_ copies/ml) and the lowest CD4 count were observed in the 20 patients with anti-TB and anti-infection therapies. The 357 cases had been receiving HAART treatment for average 19.3 months (m) (ranging from 1 week to 68.6 m), while 35.9% (128/357) of them had been treated for less than 6 months. Among 229 patients who had been treated for longer than 6 months, 12 (5.2%) HAART-treated patients had plasma viral load higher than 200 copies/ml (indicating the clinical viral failure of HAART). The CD4 count (mean ± SD) was significantly (P<0.01) higher in patients with longer HAART treatment duration (262±169 cells/µl for 6–12 m, 335±206 cells/µl for 12–24 m, 372±201 cells/µl for 24–36 m, and 427±231 cells/µl for >36 m) than in patients with HAART less than 6 months (142±133 cells/µl).

### HIV-1 Genotyping and Drug Resistance Mutation Analysis

Of 200 samples delivered into *pol* and *env* partial gene sequence analysis, 157 samples were achieved with both *pol* and *env* sequences. In addition, 21 samples achieving with *pol* sequences but experienced an amplification failure by *env*-specific RT-PCR, another 9 achieving *env* sequences but failed to be amplified by *pol*-specific RT-PCR, and the remaining 13 failed to be amplified by both *env*- and *pol*-specific RT-PCRs. The characteristics of these 157 participants, including their ages, risk factors, occupations, and treatment statuses were similar to that of all 508 patients (Fig. 1b). Of 157 sequence samples analyzed, 152 (96.8%) had consistent subtypes (66.9% CRF01_AE, 14.8% CRF07_BC, 5.7% B’, 3.2% CRF08_BC, 3.2% B, 0.6% C, and 1.9% CRF02_AG), 5 (3.2%) showed distinct genotypes in *env* and *pol*. Among the 5 distinct genotypes, 3 (1.9%) were inter-subtype recombinants (CRF02_AG/CRF01_AE, CRF07_BC/CRF01_AE, and CRF08/B) and 2 (1.3%) were unique recombinant forms in *pol* (URFs) (Table 1). The characteristics of patients showing a potential inter-subtype recombinant were summarized in Table 2. No obvious geographic correlation was observed (data not shown).

**Table 1:** HIV-1 genotypes in infected individuals in Guangdong, China (cases/percentage).

Risk Factors	Heterosexual	IDU	Blood Transfusion	MSM		Unknown	TOTAL
**Identical genotypes in ** ***pol*** ** and ** ***env*** ** regions**							
CRF01_AE	56 (65.9%)	23 (71.9%)	6 (66.7%)	1 (50.0%)		19 (65.5%)	**105 (66.9%)**
CRF07_BC	14 (16.5%)	5 (15.6%)		1 (50.0%)		4 (12.9%)	**24 (15.3%)**
B'	4 (4.7%)	1 (3.1%)	2 (22.2%)			2 (6.9%)	**9 (5.7%)**
CRF08_BC	4 (4.7%)	1 (3.1%)					**5 (3.2%)**
B	2 (2.4%)					3 (10.3%)	**5 (3.2%)**
C			1 (11.1%)				**1 (0.6%)**
CRF02_AG	2 (2.4%)					1 (3.4%)	**3 (1.9%)**
**Recombinants** *							
CRF02_AG/CRF01_AE	1 (1.2%)						1 (0.6%)
CRF08_BC/B	1 (1.2%)						1 (0.6%)
CRF07_BC/CRF01_AE		1 (3.1%)					1 (0.6%)
Other URF	1 (1.2%)	1 (3.1%)					2 (1.3%)
**TOTAL**	**85 (54.1%)**	**32 (20.4%)**	**9 (5.7%)**	**2 (1.3%)**		**29 (18.5)**	**157 (100.0%)**

* Recombinants were determined by Simplot software.

MSM, men who have sex with men; MTC, mother to child transmission; IDUs, intravenous drug users; URF, unique recombinant forms.

**Table 2:** Characterizations of patients having *pol* and *env* sequences with potential recombinants.

No.	Sex	Route of transmission	HAART regimens	Months post-HAART	CD4^+^ T cells/µl	Plasma HIVRNA copies/ml	Subtype pol(simplot)	Subtype env(tree)
339	Male	Heterosexual	D4T+3TC+EFV	37.4	234	<50	CRF02_AG	CRF01_AE
203	Male	IDU	D4T+3TC+EFV	43.3	265	100000	CRF07_BC	CRF01_AE
197	Male	Heterosexual	None		181	40000	CRF08_BC	B
34	Male	IDU	AZT+3TC+NVP	26.9	308	<50	URF (A/C/A)	A
40	Male	Heterosexual	D4T+3TC+NVP	0.5	222	96	URF (CRF02_AG/CRF01_AE)	CRF02_AG

Among 94 *pol* sequences from patients under HAART, 10 (10.6%) showed resistance mutations evaluated by HIVdb algorithm. In contrast, 3 of 63 (4.8%) untreated patients showed resistance mutations evaluated by the calibrated population resistance tool [17]. Their genotypes, CD4 counts and viral load were shown in Table 3. Among the 12 HAART-treated patients with clinical viral failure (>200 copies/ml) in a treatment duration ≥6 months, only 1 of them (patient 440#) was correlated to multiple resistance mutations. In addition, patient 457# treated for 2.2 months showed a high viral load (20,000 copies/ml) and the resistance mutation M184V. The remaining 9 patients (patients 14#, 40#, 115#, 182#, 211#, 326#, 398#, 437#, and 540#) with diverse drug resistance mutations showed steadily a low viral load (<200 copies/ml) (Table 3).

**Table 3:** Drug resistance mutations in HAARV-treated and drug-naïve patients in Guangdong, China.

No.	Sex		Risk factors	Months post-HAART		Plasma HIV RNA copies/ml	CD4^+^ T cells/µl	Subtype (*pol/env*)	Drug resistance mutations			Treatment (susceptibility)
									NRTI	NNRTI	PI	
**Treated group, 11 of 101 cases with drug-resistance mutations (HIVdb program analysis)**												
457		M	Heterosexual		2.2	2.0×10^4^	77	CRF07_BC/CRF07_BC	M184V^***,^ †			TDF(S),3TC(H),EFV(S)
440§		M	Heterosexual		20.7	1.3×10^5^	142	CRF01_AE/CRF01_AE	M184V^***^, V75S‡	K101Q*, Y181C*,†,G190A^**^		AZT(S),3TC(H),EFV(H)
437§		M	Heterosexual		35.4	<50	303	CRF01_AE/CRF01_AE	V75S*, M184V^***^	K101Q*, Y181C *, G190A^**^		D4T(L),3TC(H),EFV(H)
182		M	Heterosexual		14.6	<50	307	CRF01_AE/B	M184V^***,^ †, N348I‡	K103N^***^		D4T(S),3TC(H),Kaletera(S)
398		M	Heterosexual		27.1	<50	290	CRF01_AE/CRF01_AE	M184MV^***,^ †			D4T(S),3TC(H),EFV(S)
211		M	Heterosexual		41.7	<50	316	CRF01_AE/CRF01_AE	A62V^#^, K65R^**,^ ††Q151M(V75I)^***^	K101E*, G190A^**^Y181C(V179F)*		AZT(I),3TC(I),EFV(H)
326		M	IDU		68.3	<50	223	CRF01_AE/B	T215Y(M41L,L210W,D67N)^***^, K219R^#^	K103N(K238T)^***^ G190A^***^	L33F‡	D4T(H),3TC(L),NVP(H)
40		M	Heterosexual		0.5	96	222	URF/CRF02_AG			L10V‡, G73C‡	D4T(S),3TC(S),NVP(S)
14		M	MSM		1	59	334	CRF01_AE/CRF01_AE	T215IT^#^			AZT(L),3TC(S),NVP(S)
115		M	IDU		2	<50	39	CRF01_AE/CRF01_AE		V179D*		D4T(S),3TC(S),NVP(PL)
540		F	Heterosexual		56.5	<50	239	CRF02_AG/CRF02_AG		V179E*		AZT(S),3TC(S),NVP(PL)
**UNTREATED group, 3 of 68 cases with SDRM (2009)**												
372		M	Unknown		None	1.1×10^4^	375	CRF01_AE/CRF01_AE	K70R, V75M, M184V, K219N	K101E, Y181C, G190A		
512		F	Heterosexual		None	1.0×10^3^	451	B/B			M46I	
373		F	Unknown		None	<50	352	B/B	M41L, T215E			

* , **,*** indicate the low-level, intermediate or high-level resistance to HAART drugs administered, respectively.

‡ indicates the existence of drug-resistance mutations while no corresponding drugs were administered to the patient.

† indicates the reduced susceptibility to one HAART drug administered to the patient, while increase the susceptibility to the other HAART drugs simultaneously.

†† indicates the hyper-susceptibility to AZT.

§ HIV-1 of patients 440 and 437 presented in the same transmission chain as identified by the PhyML analysis.

# indicates the mutations occurring with other drug-resistance mutations, while their effect on susceptibility is uncertain.

M, Male; F, Female; IDUs, intravenous drug users; MSM, Men having sex with men; SDRM, surveillance drug resistance mutation; NRTI, nucleoside reverse transcriptase inhibitors; NNRTI, non- nucleoside reverse transcriptase inhibitors; PI, protease inhibitors; TDF, tenofovir; 3TC, lamivudine; AZT, zidovudine; d4T, stavudine; DDI, didanosine; NVP, nevirapine; EFV, efavirenz; Kaletera, combination of lopinavir and ritonavir; S, susceptible; H, high-level resistance; I, intermediate resistance; L, low-level resistance; and PL, potential low-level resistance.

### Identification of Transmission Clusters

**Figure 2:**
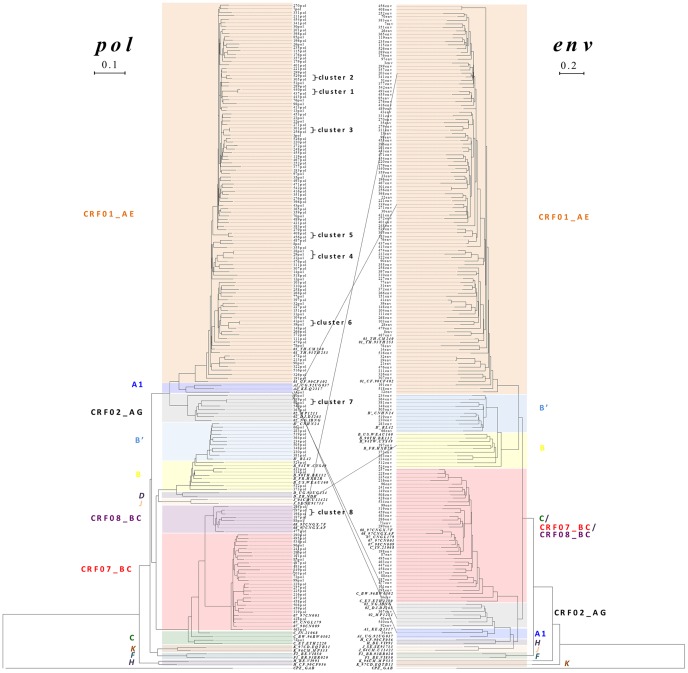
Phylogenetic tree (maximum likelihood) and genotypes of HIV-1 in Guangdong’s patients. Continual lines connected the inter-subtype recombination sequences of *pol* (left) and *env* (right) trees from the same patient.

Of 157 *pol* sequence samples analyzed, only 17 (10.8%) sequences segregated into 8 tiny transmission clusters defined by PhyML (Fig. 2). All clusters included the sequences from 2 patients, except one with three (cluster 4). The only transmission cluster (cluster 1) with drug resistance mutations (patients 440# and 437#) was identified as showed in Table 3. Of 8 clusters identified, 6 transmission chains had been confirmed by contact tracing (including the sex, age, habitation, occupation of patients in the same cluster), while two clusters (clusters 5 & 8) remained ambiguous: one (cluster 5) derived from one child (#456) who got HIV-1 from blood transfusion and one man (#408) who was not the source of contaminated blood supply; another one (cluster 8) derived from 2 men (patients 197# and 198#) who were reported to contract HIV-1 infection by heterosexual route in the same city (Guangzhou). The characteristics of 17 patients showing *pol* sequences within the 8 transmission clusters were summarized in Table 4. Among the remaining 140 sequences, 4 (216, 225, 228, and 257) showing a bootstrap >0.98 had a genetic distance of 0.016 while 7 sequences showing a bootstrap between 0.90 and 0.98 had a genetic distance >0.020. However, their potential transmission linkages were disapproved by contact tracing.

**Table 4:** Characteristics of patients showing pol sequences within the 10 transmission clusters identified by phylogenetic reconstruction.

Cluster	No.	Sex	Age	Prefecture/City	Route oftransmission	HAART regimens	Months post-HAART	CD4^+^ Tcells/µl	Plasma HIV RNA copies/ml	Subtype pol(simplot)	Subtype env (tree)
**1**	440	Male	33	Yangjiang	Heterosexual	AZT+3TC+EFV	20.7	142	131669	CRF01_AE	CRF01_AE
	437	Male	44	Yunfu	Heterosexual	D4T+3TC+EFV	35.4	303	<50	CRF01_AE	CRF01_AE
**2**	529	Female	26	JiangMen	Heterosexual	D4T+3TC+NVP	0.5	299	<50	CRF01_AE	CRF01_AE
	305	Male	34	JiangMen	IDU	D4T+3TC+EFV	1.2	125	<50	CRF01_AE	CRF01_AE
**3**	301	Female	35	Yangjiang	Heterosexual	None		264	<50	CRF01_AE	CRF01_AE
	256	Male	41	Yunfu	Heterosexual	AZT+3TC+NVP	17.2	425	<508	CRF01_AE	CRF01_AE
**4**	28	Male	37	Shanwei	Heterosexual	D4T+3TC+EFV	1.1	30	277	CRF01_AE	CRF01_AE
	29	Male	54	Shanwei	Heterosexual	D4T+3TC+NVP	0.2	182	1000	CRF01_AE	CRF01_AE
	21	Female	57	Shanwei	Heterosexual	D4T+3TC+NVP	1.2	237	<50	CRF01_AE	CRF01_AE
**5**	408	Male	27	DongGuan	Heterosexual	D4T+3TC+NVP	3.1	77	<50	CRF01_AE	CRF01_AE
	456	Male	10	Zhongshan	Unknown	D4T+3TC+NVP	8.8	888	<50	CRF01_AE	CRF01_AE
**6**	39	Female	37	Sichuang Province	Blood transfusion	D4T+3TC+NVP	24.6	378	<50	CRF01_AE	CRF01_AE
	34	Male	31	Yangjiang	IDU	AZT+3TC+NVP	26.9	308	<50	URF (A/C/A)	A
**7**	339	Male	39	FoShan	Heterosexual	D4T+3TC+EFV	37.4	234	<50	CRF02_AG	CRF01_AE
	92	Female	30	FoShan	Heterosexual	None		350	<50	CRF02_AG	CRF02_AG
**8**	197	Male	31	GuangZhou	Heterosexual	None		181	40000	CRF08_BC	B
	198	Male	39	GuangZhou	Heterosexual	None		176	38107	CRF08_BC	CRF08_BC

## Discussion

Until October 2011, 32,195 people were reported as HIV-1-infected individuals in Guangdong, a total of 9808 were diagnosed as AIDS and 7083 of them died (http://www.gdwst.gov.cn/a/zwxw/201111309428.html). Although NFATP has been implemented since 2002 (initially to former plasma donors and then to the whole population) [18], the overall treatment coverage for treatment-eligible population reached only 63.4% by 2009 while it remained particularly low (42.7%) in IDUs as compared to sexually infected patients (61.7%) and those infected through plasma donation and blood transfusion (80.2%) [4]. Since 18.7% infected individuals were IDUs and as high as 66.1% were unemployed or 17.9% were farmer (Fig. 1b), it was clear that people in low social-status faced a higher risk and it would be more difficult for them to access the NFATP program, thereby causing a delay in receiving the HAART treatment. The fact that new HIV-1-infected cases diagnosed mainly at an advanced stage of infection (http://www.gdwst.gov.cn/a/yiqingxx/201002147510.html) could be the principal cause of an overall high mortality in this region.

Although an emergence of drug resistant variants might account for the regional high mortality, this seams unlikely in our findings since 10 (10.6%) HAART-treated patients had drug resistance mutations to the first-line NRTIs and NNRTIs, which correlated to the clinical failure in controlling their viral loads in only 2 (2.1%) cases. Although repeated measures should be conducted to exclude the possibility that we had missed the minority resistant stains, other reasons like drug adherence might also cause the viral failure as reported previously [19]. It is worthy to note that infection with drug resistant HIV-1 variants might not necessarily cause the clinical viral failure, even if virus resistance to all administered drugs existed, some patients may maintain a low viral load (<200 copies/ml) [20]. It was intriguing that four patients (437, 182, 211, and 326) with high-level multiple drug resistance mutations had an undetectable viral load (<50 copies/ml). It seems also plausible that the replication of these viral strains showing multiple drug resistance mutations might be controlled in vivo by their CD8^+^ T cells since these viruses did replicate well in PBMCs after depleting of CD8^+^ T cells ex vivo. It could be interesting to follow up these patients to verify whether their viral loads will remain undetectable after withdrawing from HAART.

We found 12 genotypes of HIV-1 strains including 7 subtypes (B, B′, C, CRF01_AE, CRF02_AG, CRF07_BC, CRF08_BC), and 5 inter-subtype recombinants from which 3 mosaic in *pol*. CRF01_AE, subtype B and subtype C are dominant in Asia [21]. Subtype C is the most prevalent globally, which is found predominantly in India. Subtype B′ prevalence in China was thought to be founded by a single lineage of pandemic subtype B around 1985 [22]. CRF07_BC and CRF08_BC have been spreading through IDUs and are currently circulating in majority of mainland China [23], [24], [25], [26]. CRF02_AG is the predominant molecular form of HIV-1 in some western African countries [27], and also reported in China [28]. Several recombinants were also identified in our study and their full-length genomes need to be sequenced as they could potentially represent a new CRF. Although we cannot exclude the possibility that different fragments (*pol* or *env*) may be amplified from distinct HIV-1 strains in an individual patient, this probability should be very rare since any infection with dual or multiple HIV-1 quasi-species will result in an outgrowth of dominant/major HIV-1 strain during primary infection, which may be a new recombinant strain formed quickly after dual or multiple infections [29]. In fact, the genotyping results on *pol* and *env* fragments were generated from dominant sequences amplified from the major HIV-1 strain of each individual patient. The dual or multiple HIV-1 infections can be confirmed generally by clonal sequence analysis of viral quasi-species in seropositive individuals. The higher diversity of HIV-1 in Guangdong implies the multiple introductions of HIV-1 strains in such a most active world-trade region in China.

Phylogenetic analysis of viral gene sequences has successfully been used to construct direct or indirect epidemiological links in geographically defined populations with acute/primary or chronic HIV-1 infection [6], [30], [31], [32], [33]. In our study, heterosexual contact was the dominant route of HIV-1 transmission in Guangdong as reported by others [3], and 8 tiny transmission chains were identified by phylogenetic analysis of *pol*, which was also coincident with the characteristics of heterosexual transmission as reported previously by our group [7], [11]. Of 8 clusters identified, 6 heterosexual transmission chains were confirmed by contact tracing with two exceptions that both men in cluster 8 declared definitely a heterosexual route of transmission and one child (#456) in cluster 5 got HIV-1 from blood transfusion while the man (#408) in cluster 5 was not the source of contaminated blood supply. In addition, we confirmed 1 transmission chain (cluster 1) involving 2 patients infected by HIV-1 with identical drug resistance mutations. Moreover, phylogenetic transmission reconstruction may provide the evidence of dual (or super) infection. For example, patient 399 (in cluster 7), in contrast to his heterosexual partner (patient 92) who remained a pure subtype (CRF02_AG), was most likely super-infected by CRF01_AE (the dominant strain in the region), leading to a new CRF02_AG/CRF01_AE recombinant subtype. Furthermore, patient 197 (in cluster 8) were also likely super-infected by diverse strains of HIV-1 (CRF08_BC/B) (confirmed by repeated PCR and sequencing), resulting in new inter-subtype recombinants (Table 4). Thus, dual or super infections might contribute to the diversity of HIV-1 subtypes in the region.

Taken together, our findings demonstrated that HIV-1 CRF01_AE was a major subtype accounting for HIV-1-infected patients in Guangdong. Although not common, transmission of drug resistant strains did exist. The major risk factors for HIV-1-related mortality were most likely not receiving HAART and having a low CD4 count (<50 cells/µl) when first declared eligible for treatment as reported earlier [4]. Given that about 323,252 (43.7%) of the estimated 740,000 HIV-infected individuals living in China at the end of 2009 were identified [34], it is possible that the high proportion (>50%) of undiagnosed people with advanced chronic HIV-1 infection (associated with high viral loads) might be a major source accounting for the current outbreak of sexually acquired HIV-1 transmission in China. Thus, there is an urgent need for earlier HIV-1 diagnosis allowing better access to treatment so as to decrease the HIV-1-related mortality and limit the source of the sexually transmitted virus.
