# Correction: Molecular Epidemiology of Human Immunodeficiency Virus Type 1 in Guangdong Province of Southern China

**DOI:** 10.1371/annotation/351db299-a67a-48e0-88c6-4d9fba50f9a4

**Published:** 2013-01-23

**Authors:** Song Chen, Weiping Cai, Jinyang He, Nicole Vidal, Chunhui Lai, Weizhong Guo, Haolan He, Xiejie Chen, Linsheng Fu, Martine Peeters, Eric Delaporte, Jean-Marie Andrieu, Wei Lu

The third author's name was spelled incorrectly.

The correct name is: Jinyang He. 

