# Correction: Molecular Epidemiology of Human Immunodeficiency Virus Type 1 in Guangdong Province of Southern China

**DOI:** 10.1371/annotation/8995647b-ecd7-4a62-9365-26230ee62434

**Published:** 2013-05-10

**Authors:** Song Chen, Weiping Cai, Jingyang He, Nicole Vidal, Chunhui Lai, Weizhong Guo, Haolan He, Xiejie Chen, Linchun Fu, Martine Peeters, Eric Delaporte, Jean-Marie Andrieu, Wei Lu

The ninth author's name was spelled incorrectly.

The correct name is: Linchun Fu. 

